# Peptide Induced Crystallization of Calcium Carbonate on Wrinkle Patterned Substrate: Implications for Chitin Formation in Molluscs

**DOI:** 10.3390/ijms140611842

**Published:** 2013-06-04

**Authors:** Anindita Sengupta Ghatak, Marcus Koch, Christina Guth, Ingrid M. Weiss

**Affiliations:** 1 Program Division “Biomineralization”, INM—Leibniz Institute for New Materials gGmbH, D-66123 Saarbruecken, Germany; E-Mail: christina.guth@inm-gmbh.de; 2 Program Division “Innovative Electron Microscopy”, INM—Leibniz Institute for New Materials gGmbH, D-66123 Saarbruecken, Germany; E-Mail: marcus.koch@inm-gmbh.de

**Keywords:** wrinkle substrate, peptide, crystal, pH, composites, chitin synthase, mollusc shell

## Abstract

We here present the nucleation and growth of calcium carbonate under the influence of synthetic peptides on topographically patterned poly(dimethylsiloxane) (PDMS) substrates, which have a controlled density of defects between the wrinkles. Experiments with two lysine-rich peptides derived from the extracellular conserved domain E22 of the mollusc chitin synthase *Ar*-CS1, AKKKKKAS (AS8) and EEKKKKKES (ES9) on these substrates showed their influence on the calcium carbonate morphology. A transition from polycrystalline composites to single crystalline phases was achieved with the peptide AS8 by changing the pH of the buffer solution. We analyzed three different pH values as previous experiments showed that E22 interacts with aragonite biominerals more strongly at pH 7.75 than at pH 9.0. At any given pH, crystals appeared in characteristic morphologies only on wrinkled substrates, and did not occur on the flat, wrinkle-free PDMS substrate. These results suggest that these wrinkled substrates could be useful for controlling the morphologies of other mineral/peptide and mineral/protein composites. In nature, these templates are formed enzymatically by glycosyltransferases containing pH-sensitive epitopes, similar to the peptides investigated here. Our *in vitro* test systems may be useful to gain understanding of the formation of distinct 3D morphologies in mollusc shells in response to local pH shifts during the mineralization of organic templates.

## 1. Introduction

Biologically controlled mineralization [1] is one of the most fascinating areas of research in material science as it has the unique ability to control the crystal polymorphism, orientation, and the hierarchical patterns of inorganic materials. Biominerals are formed as composite materials comprising both inorganic minerals and organic macromolecules [2]. For example, some of the most fascinating biological materials in nature are the mollusc shell and nacre. These biominerals contain approximately 95% CaCO_3_ and 5% biomacromolecule-like soluble and insoluble proteins and polysaccharides. Chitin is one of the key components needed for mollusc shell and nacre formation [3–8]. Chitin plays a major role in the mineralization process by forming the framework for other macromolecules and, in some cases, it even contributes to the control of crystal polymorphism [9]. The hierarchically organized, layered ultrastructures of mollusc shells exhibit a mechanical strength about 30 times higher than that of the inorganic CaCO_3_ they are made of [10]. Researchers have been studying various aspects of their hierarchical structures, functionality, and mechanical properties, aiming to mimic this composite material, which has diverse technological applications on account of its supreme optical, electrical, magnetic, and mechanical properties [11–17]. Central to all these applications is the understanding of the process of mineralization and crystal growth.

**Figure 1:**
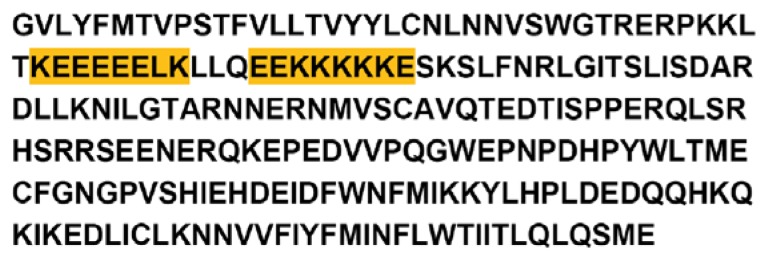
Partial sequence encoding an extracellular domain of *Ar*-CS1, the myocin chitin synthase of the bivalve *Atrina rigida*.

There is a unique transmembrane enzyme involved in the hierarchical formation of mollusc shells: the so-called mollusc chitin synthase. It’s importance was noticed when larval shells were formed in the presence of Nikkomycin Z, a drug acting as a competitive inhibitor of chitin synthesis [18]. The ordered formation of shells failed when mollusc chitin synthase was only partially blocked, and even the formation of mineral crystals was disturbed. These experiments suggested that the enzymatic synthesis of chitin was somehow coupled to the inorganic mineralization. In fact, one of the extracellular domains of the fully sequenced mollusc chitin synthases of three different bivalve molluscs (*Atrina*, *Mytilus*, *Pinctada; Ar*-CS1 [Acc.No. DQ081727], *Mg*-CS1 [Acc.No. EF535882], and *Pf*-ChS1 [Acc.No. BAF73720]) is highly conserved with respect to acidic and basic amino acid residues (Figure 1).

The role of these residues has, so far, not been investigated in detail. In the present study, we assumed that the transmembrane chitin synthases are located at the growth front of shell formation. We further assumed that the pH of calcium carbonate precipitation is certainly above the pKa values of Asp (D) and Glu (E). The amino acid residues, which eventually undergo a dynamic transition between a charged and uncharged state at the conditions of shell formation, are basic amino acids such as lysine (K) and arginine (R) [19]. In fact, the extracellular loop of chitin synthase (Figure 1) contains a unique poly-lysine motif with five lysines, which are flanked by glutamic acids, two of which are located *N*-terminal and one is *C*-terminal of the 5× Lys (5× K) motif. The fact that this motif appears only in mollusc chitin synthases, but not in other chitin synthases, suggests that it influences the regulation of chitin formation as a function of mineralization [20]. As precipitation and dissolution of mineral phases never occurs without any change in pH, charged *vs.* uncharged epitopes would be an ideal trigger to coordinate the formation of chitin and mineral in the mollusc shell [21,22].

Recently, patterned surfaces have attracted attention in the field of biomimetic mineralization. They control crystal nucleation [23–28] and also influence crystallographic orientations [29]. Here, we were particularly interested to see whether the interaction of biomolecules with calcium carbonate crystallization is better controlled on wrinkled patterned surfaces.

**Figure 2:**
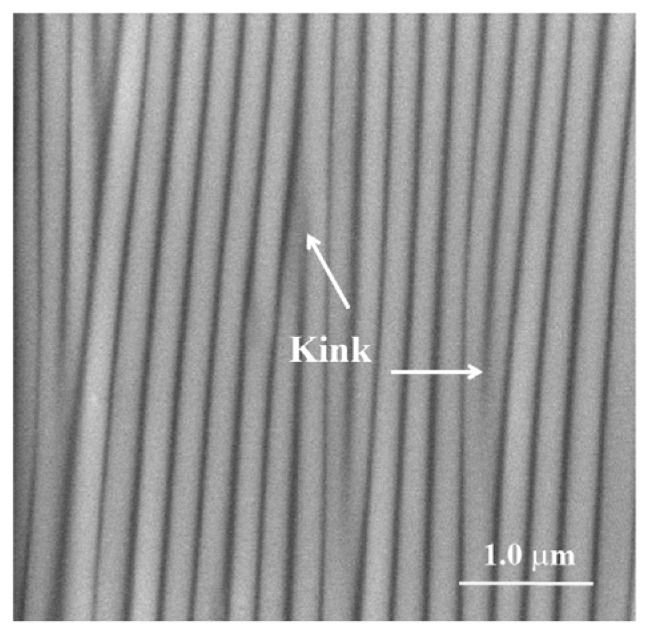
Scanning electron microscopy (SEM) image of the stretch induced surface wrinkles generated on the poly(dimethylsiloxane) (PDMS) substrate. The defects are visualized here as the sharp kinks between neighboring wrinkles. The SEM image was taken at 10 KeV, spot size 3 (1.7 nm), 50.000× magnification.

In previous work Ghatak and her colleague showed that the presence of wrinkled surfaces resulted in controlled nucleation [30]. They also showed the generation of large single-protein crystals, reaching sizes of 180 μm, whereas the typical size obtained without wrinkled patterns is around 50 μm [30]. There are cases where protein crystals were obtained only on wrinkled substrates but not on flat ones [31]. They further investigated how the defect density increases with the increase in stretching ratio (λ = final length/initial length). Protein crystal size attained maximum at λ = 1.4, beyond which the crystal size decreases [30]. The kink shaped defects may act as precursors for lowering energy barriers, which have maximum control in influencing the nucleation and the crystal morphologies. If defects of controlled size, shape, and number density on a poly(dimethylsiloxane) (PDMS) substrate promote nucleation of protein crystal formation, the above mechanism should be valid for the nucleation of CaCO_3_ as well. Figure 2 shows the shape and distribution of kink shaped defects in a typical sample of a wrinkled substrate.

For all *in vitro* experiments mimicking biomineralization it is most crucial to achieve very sensitive control over calcium carbonate precipitation. It was therefore an important goal of this study to see whether this can be achieved by taking advantage of wrinkle substrate concepts [32]. Due to the simplicity of this well-established method, one can easily generate reproducible wrinkle patterns of a periodicity of about 200 nm. A thin strip of PDMS film is first subjected to uni-axial stretching, then to plasma oxidation of its surface in order to generate a thin crust of silica, and afterwards the stretched film is released to generate wrinkles via difference in deformability of the hard top crust on a soft substrate. Using these wrinkled substrates we have investigated how the different synthetic peptides derived from an extracellular chitin synthase domain influence the nucleation of calcium carbonate.

In order to design the synthetic peptides we have compared the multiple sequence alignment of the chitin synthase amino acid sequences of *Lottia gigantea* (*Lg*), *Atrina rigida* [*Ar*], *Mytilus galloprovincialis* [*Mg*], and *Pinctada fucata* [*Pf*] [33–35]. So far, database analysis of the three dimensional structures available in the Protein Data Bank (PDB) using the program blastp [36] did not yield any epitopes with similarities to the designed peptides.

In this paper we show that the wrinkle-pattern substrate (Figure 2) influenced the nucleation of calcite crystals. These results provide the first evidence that wrinkle substrates obtain a high level of control over calcium carbonate crystallization, a prerequisite for studying the functional roles of organic additives in complex biomineralization systems.

## 2. Results and Discussion

In the present work, we compared the influence of two basic peptides (AS8 & ES9), each comprising uncharged and charged terminal residues on the nucleation of calcium carbonate (Table 1). Although the central residues (KKKKK) are the same in these two peptides, interestingly they behave quite differently due to the presence of negatively charged glutamate residues. Figure 2 depicts a scanning electron microscopy image of the wrinkle-patterned substrate with a periodicity of ~0.2 μm. Sharp kinks were formed between the two neighboring wrinkles, acting as nucleation sites. The basic idea for our experiment was to achieve approximately equal densities of peptide ions, which are in equilibrium with their respective uncharged peptides. Each peptide carries multiple charges depending on the solutions used in the crystallization experiments, each at a particular pH. We therefore calculated the molarities of the peptides accordingly at three different pH values (pH 7.75, pH 8.2, and pH 9.0) by using an approximation based on the Henderson-Hasselbalch equation (see Supplementary Information SI_4 in [20] and webtool “livinig graph” in [19]) (Table 2). In addition to the basic peptides, we examined the influences of both acidic and basic peptides together on the mineralization of calcium carbonate (Table 1).

### 2.1. Comparative LC-PolScope Analyses of CaCO_3_ Crystals in the Presence of Basic Peptides

**Figure 3:**
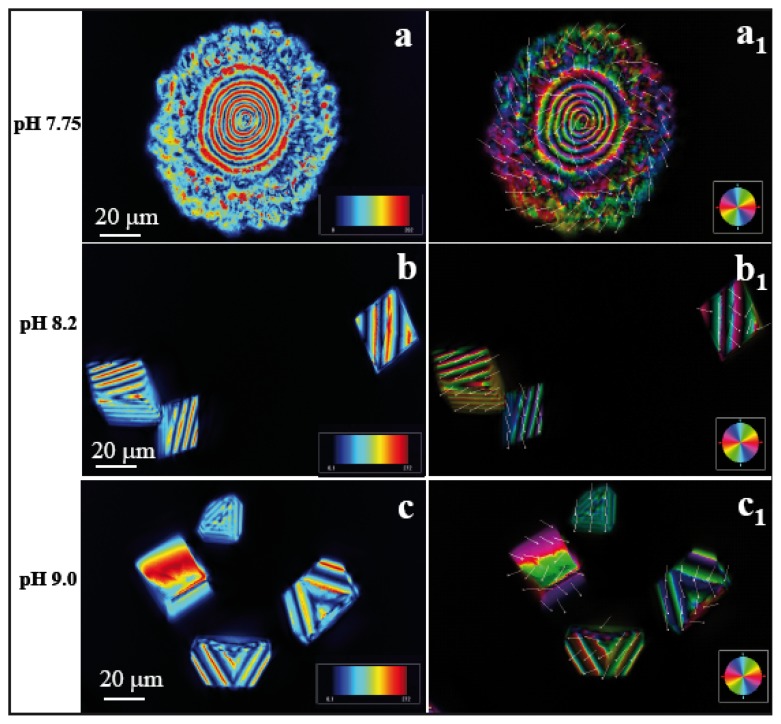
LC-PolScope images of CaCO_3_ crystals grown in presence of peptide AS8 (pI 10.6) at pH 7.75, pH 8.2, and pH 9.0, respectively, in the quantitative birefringence mode showing the retardance and orientation images. Scale bar: 20 μm. The experiment shows a smooth transition from polycrystalline to single crystal morphology obtained by changing the pH value. (**a**,**a_1_**) Peptide AS8 at pH 7.75, *c* = 9.66 mM; (**b**,**b_1_**) Peptide AS8 at pH 8.2, *c* = 9.09 mM; (**c**,**c_1_**) Peptide AS8 at pH 9.0, *c* = 6.13 mM.

We present here the results from experiments with CaCO_3_ crystals in presence of peptide AS8 on the patterned wrinkle substrate (Figure 2). Figure 3a,a_1_ represent the images taken in LC-PolScope for the analysis of birefringent retardance and orientation images, respectively at pH 7.75 ([Ca^2+^] = 10 mM, AS8 = 9.66 mM). The dark red area has a retardance value of 272 nm while the dark blue has 0.1 nm. In order to differentiate pure and composite materials, we checked the retardance of pure CaCO_3_ crystals of calcite on the wrinkle pattern obtained without any peptide, which has retardance values on the dark red area 120 nm and black/darkblue area 0.1 nm at pH 7.75. The polycrystalline morphology of the calcite/peptide composite particles showed alternating red and blue concentric circles, in this case with a periodicity of ~3.5 μm within a nearly circular area with a radius of ~20 μm (Figure 3a). The presence of N and Ca signals in the energy dispersive spectroscopy (EDS) spectra (see also Section 2.2) confirms that polycrystalline assemblies are made of calcite/peptide composites. A marked difference of the crystal morphology was observed at pH 8.2 ([Ca^2+^] = 10 mM, AS8 = 9.09 mM). Here, a transition from polycrystals to the rhombohedral morphology (Figure 3b) yielded calcite particles of ~30μm in size. The orientation image (Figure 3b_1_) indicates that the crystals cover a wide range of crystallographic orientations. In this context, a recent report by Schneider *et al.* [38], which investigated the birefringence of calcite and aragonite structures in a 60 μm thin section of *Haliotis* shell, pointed out that differently oriented calcite crystals certainly yield different retardance values, depending on the orientation of the c-axis orientation, and that the presence of both organic and inorganic phases in small sampling volumes would be difficult to interpret [39,40]. By further increasing the pH to 9.0 ([Ca^2+^] = 10 mM, AS8 = 6.13 mM), the experiment yielded a modified form of rhombohedral morphology (Figure 3c). In the supporting information we present the representative LC-PolScope images of calcite particles generated on flat, wrinkle-free substrate (Figure S1). All crystals were identified as 100% calcite at different pH values, as confirmed by Raman imaging microscopy, and the spectra are shown in the supporting information (Figure S2).

**Figure 4:**
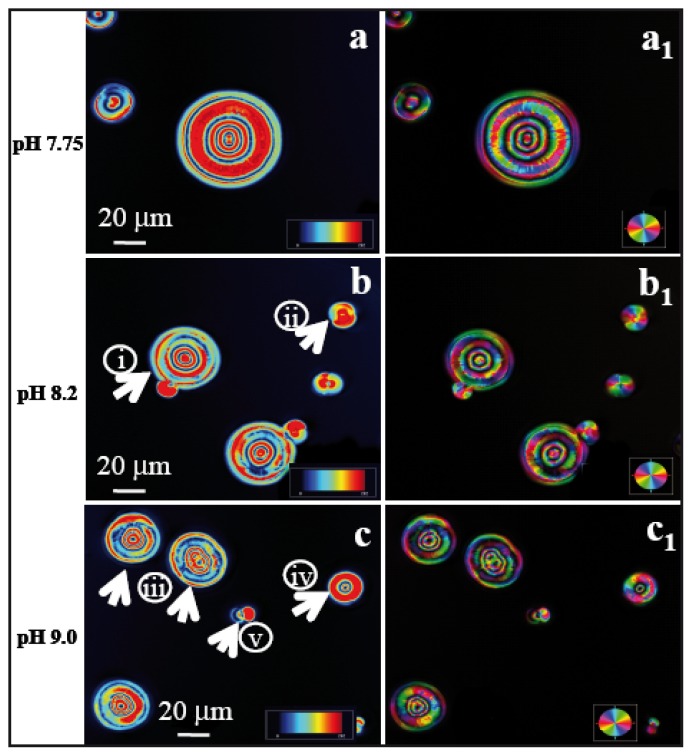
LC-PolScope images of CaCO_3_ crystals grown in presence of peptide ES9 (pI 9.41) at pH 7.75, pH 8.2, and pH 9.0. Birefringence data show the retardance and orientation images left and right, respectively. Scale bar: 20 μm. (**a**,**a_1_**) Peptide ES9 at pH 7.75, *c* = 10 mM; (**b**,**b_1_**) Peptide ES9 at pH 8.2, *c* = 10 mM; (**c**,**c_1_**) Peptide ES9 at pH 9.0, *c* = 10 mM. The numbers in figures (**b**) (i, ii) and (**c**) (iii–v) indicate the different morphologies of CaCO_3_ crystals. Note that the outer ring of polycrystals shows retardance values in the blue range (arrows).

The birefringence retardance and orientation images taken in LC-PolScope for CaCO_3_ crystals in the presence of peptide ES9 are shown in Figure 4. While all the crystals are polycrystalline in nature, different morphologies have been observed for varying the concentration of peptide ions. Crystals are assembled into concentric circles with a diameter of ~20 μm at pH 7.75 as observed in Figure 4a. In contrast, calcite/peptide composite crystallized at two entirely different morphologies at pH 8.2 (Figure 4b). The particles were identified as calcite by Raman microscopy and the spectra are shown in Figure S3 of the supporting information. Similar morphological changes were observed also at pH 9.0 as shown in Figure 4c. It is clear that the patterned substrate plays an important role here in order to induce different forms of crystalline assemblies. Calcite particles formed on flat wrinkle free substrate did not show such a defined morphological form (supporting information, Figures S1 and S3).

It is important to note that birefringence retardance and orientation images taken in LC-PolScope depend on the thickness and orientation of the sample as well as the purity of birefringent crystals. The color scales in Figures 3 and 4 are dependent on the azimuth of the optic axis. For calcite, a negatively birefringent, uniaxial crystal (*n*_e_ − *n*_o_ = −0.174), the optic axis is oriented perpendicularly to the slow axis of birefringence. The geometric relationship between the optic axis orientation and the direction of a ray passing through calcite crystals of different orientations has so far only been determined for homogeneously thin films using microlens arrays [41]. The LC-PolScope mapping of a thin section of a mollusc shell showed that the orientation of composite calcite crystals can be monitored, whereas at smaller scales, organic interfaces with different refractive index within composite aragonite crystals, the LC-PolScope maps are extremely hard to interpret [38]. For interpreting the regular patterns observed in the maps for calcite rhombohedra (Figure 3b,c) one has to take into account that the tilted crystal surfaces and the change in physical path length determine the retardance measured in a given aperture point in focus. One can assume here that the physical path length and the orientation of the optical axis are interrelated and therefore account for the regular pattern. The presence of highly regular organic defects, which hypothetically could also explain such patterns, seems less likely (compare also [21]). In contrast, the spherical crystals in Figure 4 are all polycrystalline in nature. It can be concluded from the degree of regularity of the concentric rings in the LC-PolScope maps that frequent adsorption and desorption of organic peptides at the interface introduced precipitation and ion depletion in different orientations and therefore prevented the formation of single crystals. This hypothesis is supported by the observation that the outer ring of polycrystals frequently shows retardance values in the lower, blue range (Figure 4, arrows).

### 2.2. Comparative Scanning Electron Microscopy Analyses of CaCO_3_ Crystals in the Presence of Basic Peptides

**Figure 5:**
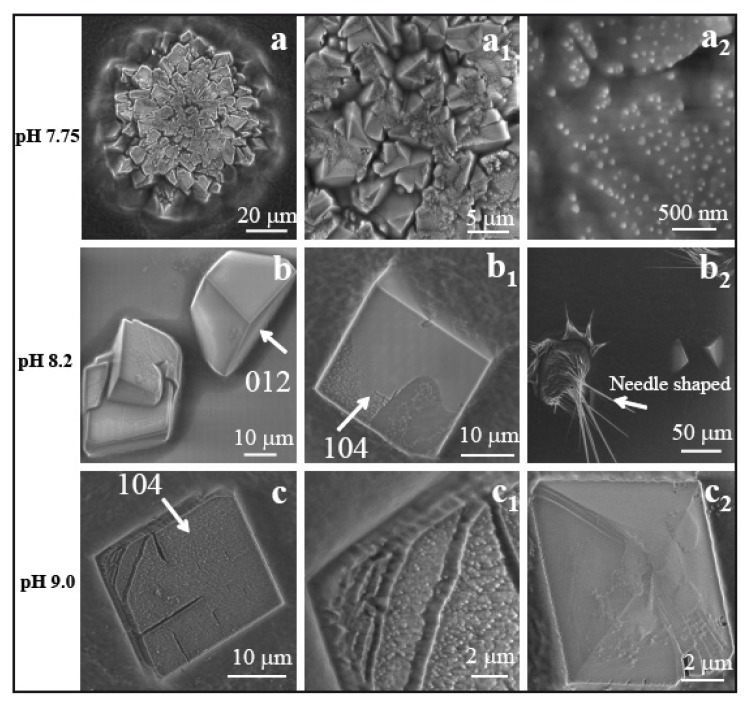
SEM image of CaCO_3_ crystal assembly in presence of AS8: (**a**) polycrystalline spherical assembly at pH 7.75; (**b**–**b_2_**) rhombohedral crystal of calcite in two different orientations: (**b**) (012) and (**b_1_**) (104); (**b_2_**) needle shaped peptide crystals at pH 8.2; (**c**, **c_1_**) calcite crystal with distorted face and corners observed at pH 9.0. The SEM images were taken at 10 keV (**c**, **c_1_**: 5 keV), spot size 3 (1.7 nm; **c**, **c_1_**: 2.1 nm). Magnifications: (**a**) 2000×; (**a_1_**) 10000×; (**a_2_**) 100000×; (**b**) 4000×; (**b_1_**) 5000×; (**b_2_**) 500×; (**c**) 5000×.

Figure 5a–a_2_ depict scanning electron microscopy (SEM) images of multicrystalline assemblies of calcite particles grown into a characteristic spherical shape on nano wrinkled PDMS substrate at a pH of 7.75, with the influence of peptide AKKKKKAS. The growth mode may depend on the peptide side chains, which carry positive charges from the NH_3_^+^ group of lysine (K). Therefore, each molecule can interact with the negatively charged HCO_3_^−^ through ionic interactions. The calculated concentration of charged peptides is comparatively high at pH 7.75, and amounts to 1.931 × 10^−8^ mol in 2 μL. This could as well explain the compact polycrystalline assembly. In the crystallization drop of 4 μL, the final density of peptide ions was as follows: 0.9655 × 10^−8^ mol for pH 7.75, 0.909 × 10^−8^ mol for pH 8.2, and 0.613 × 10^−8^ mol for pH 9.0. The energy dispersive spectroscopy (EDS) technique detects the peak for nitrogen and calcium in the map as observed in Figure S4a of the supporting information. The presence of nitrogen atoms in the crystal confirms that the particles grow as peptide/Ca composite materials. By increasing the pH, the available ion density is less in the solution, e.g., at pH 8.2 free peptide ions are 1.818 × 10^−8^ mol in 2 μL of volume, which resulted in a different morphology of calcite. Figure 5b,b_1_ depict the two phases of rhombohedral calcite crystals grown on wrinkle-patterned substrate. This type of crystal was not found on the wrinkle free PDMS substrate. Another interesting observation here is that we found thin needle shaped peptide crystals (Figure 5b_2_). The EDS spectra of the composite crystals consisting of peptides and calcium carbonate are presented in the supporting information (Figure S4b). With further increase in pH to 9.0, the peptide ion density reduced to 1.226 × 10^−8^ mol in 2 μL of volume. The particles observed at this pH value were distorted at their faces and corners with a basic rhombohedral form (Figure 5c,c_2_). A high magnification image suggested that the small distinct crystalline units were observed at the face (Figure 5c_1_).

**Figure 6:**
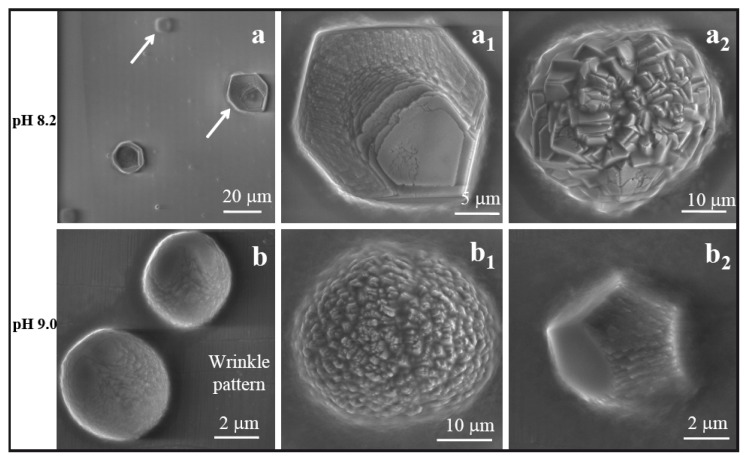
SEM images of CaCO_3_ in the presence of ES9 at pH 8.2 and 9.0. (**a**) For pH 8.2, two different forms of layer-like structures are shown: (**a_1_**) rhombohedral; (**a_2_**) spherical. (**b**) Compact spherical form; (**b_1_**) a layer like polycrystalline structure and; (**b_2_**) a new type of hexagonal crystal were obtained at pH 9.0. The SEM images were taken at 10 keV, spot size 3 (1.7 nm). Magnifications: (**a**) 2000×; (**a_1_**) 10000×; (**a_2_**) 5000×; (**b**) 20000×; (**b_1_**) 5000×; (**b_2_**) 20000×.

The peptide EEKKKKKES (ES9) has both positively (K) and negatively charged (E) residues. During the mineralization process at various pH values, different types of electrostatic interactions are possible, which subsequently controls the morphological changes of calcium carbonate: (i) the possibility of stacking interactions between the ES9 molecules; (ii) the positively charged NH_3_^+^ group of lysine side chains interact with the negatively charged HCO_3_^−^ groups and (iii) the negatively charged COO^−^ group of glutamic acid (E) side chains interact with the positively charged divalent Ca^2+^ ions. Figure 6a–a_2_ depicts two types of particles produced at pH 8.2. For example, a new type of hexagonal shaped polycrystalline assembly exhibited a layer like structure as shown in a magnified image (Figure 6a_1_) and the particles were quite flat. The second type of particles as observed in Figure 6a_2_ also had a layer like structure assembled into a spherical form. By increasing the pH to 9.0 three different type of morphologies exhibited into the solution. One of them was a compact spherical form of CaCO_3_ as appeared in Figure 6b; the other one was layer-like polycrystalline structure assembled into a spherical form with diameter ~15 μm (Figure 6b_1_), which is similar to the crystal obtained at pH 8.2. The third one showed a new type of hexagonal form in which one face of the hexagon looked like assemblies of CaCO_3_, which appeared to be made of small platelets (Figure 6b_2_).

### 2.3. LC-PolScope Analyses of CaCO_3_ Crystals in Presence of both Basic and Acidic Peptides

**Figure 7:**
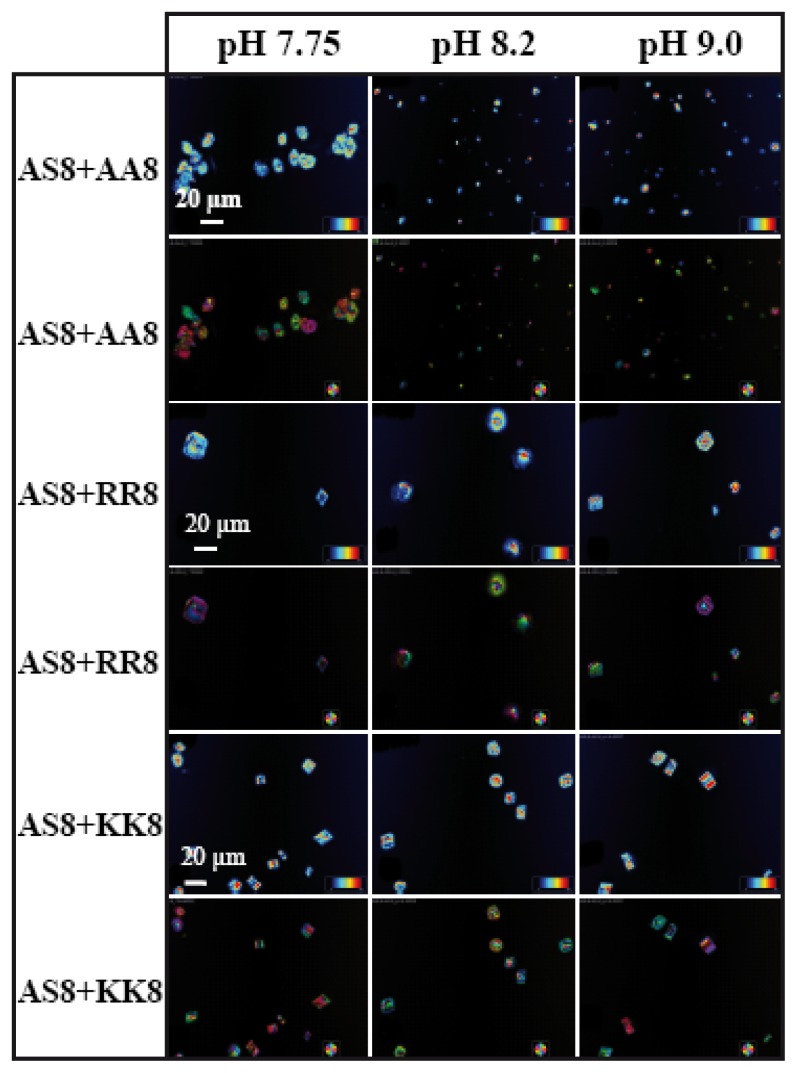
LC-PolScope images showing the birefringent retardance (1st, 3rd, 5th row) and orientation (2nd, 4th, 6th row) of CaCO_3_ crystals grown in presence of equimolar amounts of basic peptide AS8 (pI 10.6) and three acidic peptides AA8 (pI 3.51), RR8 (pI 4.33), KK8 (pI 4.33) on wrinkle patterned substrate at three different pH values. Peptide stock solutions at different pH values were: 9.66 mM (each AS8 & AA8/RR8/KK8) at pH 7.75; 9.09 mM (each AS8 & AA8/RR8/KK8) at pH 8.2; 6.13 mM (each AS8 & AA8/RR8/KK8) at pH 9.0. Note that the final peptide concentrations during crystallization were 3.22 mM, 3.03 mM, and 2.04 mM for each peptide. Scale bar: 20 μm.

**Figure 8:**
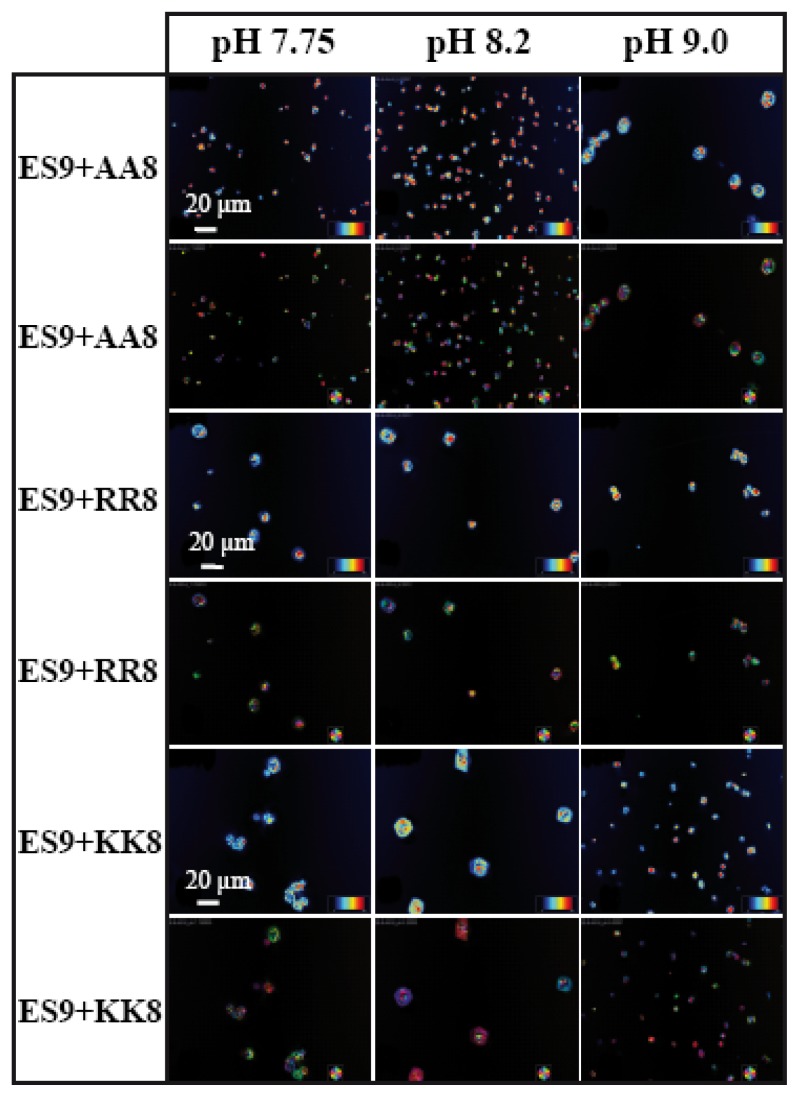
CaCO_3_ crystals grown in presence of basic peptide ES9 (pI 9.41, *c* = 3.33 mM) and three acidic peptides AA8 (pI 3.51; *c* = 3.33 mM), RR8 (pI 4.33; *c* = 3.33 mM), KK8 (pI 4.3; *c* = 3.33 mM) on wrinkle patterned substrate at three different pH values. Peptide stock solutions were 10 mM each. Images were taken in LC-PolScope mode showing the birefringent retardance (1st, 3rd, 5th row) and orientation (2nd, 4th, 6th row). Note that the switch in morphology occurs between pH 8.2 and pH 9.0 for all peptide combinations. Scale bar: 20 μm.

The above results have explained the fact that two synthetically designed basic peptides have a strong influence on the CaCO_3_ morphology at three different ionic densities, which has prompted us to examine the influences of acidic and basic peptides together on the mineralization process. Therefore, we performed six different experiments, which included several combinations of basic and acidic peptides. These are AS8 with AA8; AS8 with KK8; AS8 with RR8; ES9 with AA8; ES9 with KK8; and ES9 with RR8 (Table 1). We have considered equal molarities of both the peptides used at a particular pH value. A volume of 2 μL of acidic with 2 μL of basic peptides was used, along with 2 μL of 10 mM CaCl_2_·2H_2_O during the crystallization. It is hypothesized that the positively charged central part of one peptide ion (5 × K) could neutralize to the negatively charged central part of the other peptide ion (5 × E) through ionic interactions. In this case, the assay would investigate mainly the effect of the terminal residues: A/A, A/K, A/R in the first set of experiments, and E/A, E/K, and E/R in the second. In Figures 7 and 8 we present the LC-PolScope images for the analysis of birefringent retardance, and orientation images taken from all six combinations of peptides at three different pH values. While AS8 and AA8 together do not produce any defined shape of calcite crystals (Figure 7), however, very few rhombohedral crystals have been observed with the influence of peptides AS8 and RR8. Interestingly, the rhombohedral morphology has been dominated fully by the combination of AS8 and KK8, which suggests that among the charged amino acids, lysine has strong influence for interaction with the inorganic salt.

Similar experiments have been performed with the chitin synthase-type of basic peptide, ES9 (Figure 8). In this case, the negatively charged terminal group (E) has some influence during mineral-peptide interactions. Interestingly, only the combination of ES9 and chitin synthase-type of acidic peptide KK8 has a tendency to stabilize larger crystals (~20 μm) into the rhombohedral form at pH values 7.75 and 8.2 (Figure 8).

At this stage it remains unclear whether the dimerization/agglomeration or conformational changes of peptides in solution influence the driving force for the observed differences in the mineral morphology. Any random adsorption of single or agglomerated peptides along the line defects of the PDMS substrates would not explain the nucleation of calcite crystals in distinct spots as observed here. We can, however, not exclude the possibility that peptides are adsorbed within the wrinkled defects and therefore contribute to calcite nucleation to some extent. In a few cases, peptide crystals without the Raman signature for calcium carbonate were obtained. These were large crystals or crystal aggregates of approximately 50–100 μm in size. Some of them (AS8) seemed to originate from one distinct spot, therefore not necessarily relating to the density of kinks in the wrinkle pattern. The multi-crystalline assembly of ES9, however, suggested that some peptides are more likely to represent substrate topography in their nucleation pattern. In the natural system of mollusc shells, the acidic and basic peptides may act together on probing the mineralization process. It is very unlikely that they act directly in the center of nucleation and crystal growth, since organic matrices such as chitin are preformed and nucleation is induced at a later stage. Once mineral surfaces exist, chitin synthases could adsorb there via these peptides. This would certainly affect the orientation and patterning of multiple chitin synthases and subsequent polymer deposition. A pH-dependent cycling mechanism, especially as a function of crystal growth as suggested by the experiments reported here, is appealing to understand how the mollusc chitin synthase works.

### 2.4. Implications for Chitin Synthesis in the Context of Biomineralization

The basic peptide ES9 and the acidic peptide KK8 are part of the extracellular interface of transmembrane chitin synthase (pI 9 domain, [20]). Interestingly, these peptide blocks use glutamic acid rather than aspartic acid, and lysine rather than arginine. Both, glutamic acid and lysine have a rather low tendency to incorporate into calcite crystals [42]. The extracellular domain is more likely to represent part of a biomineralization signaling network for creating higher levels of organization via alternating chitin-mineral layers [18,20,22]. The use of wrinkle patterned substrate for calcite nucleation and growth made it possible to differentiate between the effects of peptides at three different pH values. Both, the 5× Lys and the 5× Glu domains can interfere with calcium carbonate precipitation in different ways, either individually or together. As observed here, the system is very sensitive to flanking amino acids of opposite charges (Glu-Lys *vs.* Lys-Glu, EK/KE). It shows the difficulty in determining the actual molar ratio between charged and uncharged, small and large species in solution (see also Table 2 for details) and adsorbed on either the plasma-treated PDMS substrate, calcium carbonate precipitates, or both. Especially for the latter case, one must assume that a pH shift, introduced locally by mineral precipitation or dissolution, would influence the attractive and repulsive forces between extracellular domains of individual chitin synthases while floating in the membrane [20]. We currently can not predict, neither for the peptides used here, nor for the chitin synthase domain, how the secondary and tertiary structures are influenced by complementary charged epitopes and/or precipitating/dissolving calcium carbonate at different pH values. All these possibilities would have their own specific impact on the clustering of the enzymes and thus on chitin polymerization and chitin fibril assembly. The alternating mineral phases with concentric morphologies as observed here only in the case of the native peptide ES9, regardless of pH (Figure 4), suggests that this type of regulation would provide one possible control mechanism, which seems to be very robust with respect to complex environmental parameters in the shell forming compartments.

### 2.5. Broader Impact of Calcium Carbonate Crystallization on Wrinkle Substrates

Our extensive and very precise description of this relatively simple experimental preparation of wrinkle substrate and easy crystallization procedure may help to establish a new platform for biomineralization studies that would achieve better comparability between different labs. Light optical methods are, in fact, suitable to discover even super-structures in hierarchical, natural biominerals which are composed of complicated arrangements of biopolymers and minerals on different length scales and in different crystallographic orientations [38]. This paper exactly describes the limits of light optical investigations, including LC-PolScope Abrio imaging as compared to classical polarized light microscopy and high-resolution X-ray methods, such as X-PEEM [40]. Here, we assumed that the morphologies of polycrystalline arrangements obtained by crystallization on wrinkle patterns in the presence of peptides as a function of pH can be interpreted with respect to crystallographic orientations, as long as the precipitates consist of crystallographically pure mineral phases. Here, the crystalline phase was demonstrated by Raman spectroscopy to consist of a pure calcite phase without any contamination of other crystalline phases. However, one would not be able to exclude the possibility of amorphous calcium carbonate that may eventually co-precipitate with these peptides. However, this should not disturb the analysis by LC-PolScope Abrio imaging since amorphous phases would, in fact, be "invisible". In the particular case of Figure 3a, for example, one can conclude that each particular color, in which the concentric arrangements of crystals appear, indicates a specific crystallographic orientation of calcite with respect to the imaging plane. The dark blue concentric rings strongly indicate that the c-axis of calcite is perpendicular to the imaging plane in this area of the polycrystals. More exact quantification would, in principle, be possible, provided that the dimensions of each crystal layer are known. This was, however, beyond the focus of the present study.

In summary, the extremely fast and simple LC-PolScope Abrio screening method facilitates a quick and easy evaluation of crystallization experiments for high-throughput screenings of calcium carbonate precipitates, which would certainly not be possible when using conventional X-ray and electron microscopy techniques. Our combined Raman spectroscopy and LC-PolScope Abrio imaging results presented here clearly demonstrate that this unique combination of fast and reliable methods bears an enormous potential for investigating peptide-mineral interactions on much broader scales than previously achieved.

## 3. Experimental Section

### 3.1. Preparation of Wrinkle Substrate

The wrinkle patterns were generated by a conventional method of forming surface wrinkles in the soft platform of poly(dimethylsiloxane) (PDMS). Thin strips of PDMS film (thickness ~350 μm) were stretched to a desired extension ratio of λ = 1.4 (λ = final length/initial length), following which the films were exposed to radio frequency oxygen plasma for about four minutes (Diener Plasma, model: PICO, pressure ~0.03 Torr, power ~40 W). A thin crust of silicate layer formed on the PDMS surface due to the plasma oxidation, which turned the surface hydrophilic. The stretched film was then released instantaneously. This process resulted into the formation of surface wrinkles because of the mismatch in modulus of thin silicate crust and the elastic substrate (see References in [30]). Figure 2 depicts the scanning electron microscopy image of the wrinkles generated at the PDMS substrate with an initial extension ratio of λ = 1.4. In addition to the wrinkle substrate, we used the flat wrinkle free surface of PDMS as a control and made the surface hydrophilic by exposing a small degree of plasma oxidization (Diener Plasma, model: PICO, pressure ~0.03 Torr, power ~40 W, time ~1 min).

### 3.2. Sample Preparation

A 10 mM concentration of CaCl_2_·2H_2_O dissolved in GlyGly buffer was prepared at three different pH values: pH 7.75, pH 8.2, and pH 9.0. Synthetically designed basic (AS8, ES9) and acidic peptides (AA8, RR8, and KK8) (Table 1) were used for the experiments. These peptides were commercially available from GeneCust (Dudelange, Luxembourg) with purity of 99% and dissolved in double distilled water for the crystallization experiments. PDMS films were prepared on the soft and flexible platform of cross-linked poly(dimethylsiloxane) (Sylgard 184, Dow Corning Corporation, Midland, MI, USA).

### 3.3. Calcite Nucleation and Growth in the Presence of Peptides

The crystals of CaCO_3_ were grown in the presence of peptides by a vapor diffusion method. The patterned/flat PDMS film was attached to the bottom of the crystallization wells (Lab-Tek^®^ chamber, Cat. No. 77437 Nalge-Nunc Int., Naperville, IL, USA). The chamber consisted of four wells, which were isolated from each other. Each drop of the liquid was mixed with 2 μL of CaCl_2_·2H_2_O and 2 μL of peptide solution while solid NH_4_HCO_3_ was kept in a separate well. The setup was placed inside a box and sealed with parafilm to minimize the evaporation. A beaker filled with double distilled water was kept inside the box, before sealing, in order to saturate the environment with water vapor. The entire setup was placed in a controlled environment at a temperature of 20 °C.

### 3.4. Scanning Electron Microscopy (SEM)

Scanning Electron Microscopy (SEM, FEI Quanta 400 FEG; FEI Deutschland GmbH, Frankfurt, Germany) was used in low vacuum mode (*p* = 100 Pa) to analyze the wrinkle pattern of the substrate and the morphology of the CaCO_3_ crystals in detail. Secondary electron (SE) images were captured at an operating voltage between 5 and 15 KeV. Energy dispersive X-ray (EDX) mapping of calcite crystals was done using an EDAX Genesis V6.02 X-Ray spectrometer (EDAX Inc., Mahwah, NJ, USA) at different accelerating voltages.

### 3.5. LC-PolScope Microscopy

An LC-PolScope image processing system (CRI, LOT-Oriel, Darmstadt, Germany) was used for quantitative birefringence analysis at 546 nm using the standard Abrio filter. The LC-PolScope camera was mounted on a Zeiss inverted microscope (Zeiss Observer Z1, Göttingen, Germany) equipped with A-Plan 10×/0.25Ph1, LD Plan Neofluar 20×/0.4 Korr Ph2, LD Plan Neofluar 40×/0.6Korr Ph2, and Plan Achromat 63×/1.40 Oil DIC objectives. All images were obtained according to the manufacturers instructions. Background images were taken from a selected area of the transparent specimen without any biological or crystalline sample. The Abrio™ image processing software (CRI/LOT-Oriel; [43,44]) was used for quantitative data evaluation, per pixel, with respect to both, the retardance mode and the orientation of the slow optical axis (azimuth) mode as previously described [38].

### 3.6. Raman Imaging Spectroscopy

The mineralogy of the specimens was investigated at room temperature in air using a confocal LabRAM ARAMIS Vis Raman Imaging Spectrometer (Horiba Jobin Yvon, Bensheim, Germany), equipped with 100× LWD objective Olympus MPlan (Olympus, Hamburg, Germany), and a diode laser 785 nm, 80 mW (Pilot P500, Sacher Lasertechnik Group, Marburg, Germany).

### 3.7. pI Calculations

Peptide calculations were performed using the “compute pI” tool [45] and concentrations were determined based on the Henderson-Hasselbalch equation [19,20] using the tool “living graphs” [37].

## 4. Conclusions

We have studied the interaction between the model peptides and the Ca^2+^ ions on a wrinkled substrate, with relevance for understanding the mechanism of chitin/mineral formation in biomineralization. We observed that the two basic peptides, AS8 and ES9, influenced the overall mineral morphologies. We have identified that peptides with the amino acid lysine have a tendency to preserve the rhombohedral form of calcite. A transition from polycrystalline to the single crystal rhombohedral morphology was obtained with peptide AS8, while changing the pH value from 7.75 to 8.2. The peptide ES9, more similar to the native sequence of mollusc chitin synthase extracellular domains than AS8, produced robust morphologies of concentric polycrystals, regardless of pH. The role of oppositely charged amino acids, flanking the 5× Lys and 5× Glu motifs in the pI 9 domain of mollusc chitin synthases, was highlighted by the result that crystal morphologies changed only at pH 9 in the case of K (pI 10.5), and not even at pH 9 in the case of R (pI 12.0). As a prerequisite for these experiments, we have demonstrated that the mineralization of CaCO_3_ can be highly controlled on wrinkle-patterned substrate. The substrate has a reproducible density of defects, which promotes the nucleation of crystal formation. In this manuscript, we have shown that the wrinkle features at the surface, due to the ability to gain control over the morphological changes of CaCO_3_, turned out to be useful to study peptide-mineral interactions.

## Supplementary Information



## Figures and Tables

**Table 1:** Synthetic peptide sequences §.

Name	*M.W*.	Sequence	Theoretical pI ‡	Average pK_a_†
AS8	888.13	AKKKKKAS	10.60	10.4
**ES9**	1133.32	**EEKKKKKES**	9.41	9.2
AA8	918.92	AEEEEELA	3.51	3.0
**KK8**	1033.11	**KEEEEELK**	4.33	4.3
RR8	1089.14	REEEEELR	4.33	4.3

§ A, alanine; K, lysine; S, serine; E, glutamate; R, arginine. Native *Ar*-CS1 sequences in bold;

† calculated according to manufacturer’s data sheet;

‡ according to Section 3.7.

**Table 2:** Trend estimations of cationic peptide concentrations §.

	pH 7.75	pH 8.2	pH 9.0
pKa 11: [neutral]/[total]	0.001	0.005	0.01
pKa 11: [charged] ‡	999	199	99
pKa 11: [neutral]	1	1	1
AS8 = pKa ≈	10.5 10 mM (10 mM +)†	9. mM (9 mM +)	6 mM (6 mM +)
pKa 10: [neutral]/[total]	0.01	0.025	0.1
pKa 10: [charged] ‡	99	39	9
pKa 10: [neutral]	1	1	1
ES9 = pKa ~9.5	10 mM (9 mM +)	10 mM (8 mM +)	10 mM (5 mM +)
pKa 9: [neutral]/[total]	0.1	0.125	0.5
pKa 9: [charged] ‡	9	7	1
pKa 9: [neutral]	1	1	1

§ [neutral/total] ratios estimated according to http://bcs.whfreeman.com/lehninger5e/ [37];

‡ normalized to the number of uncharged, neutral molecules (on average);

† estimation of average positive charges in brackets (symbol: +); AS8~pKa 11, ES9~pKa 9.
