# Supplementary material for: Peptide Induced Crystallization of Calcium Carbonate on Wrinkle Patterned Substrate: Implications for Chitin Formation in Molluscs
Source: Int J Mol Sci. 2013 Jun 4;14(6):11842–60. doi: 10.3390/ijms140611842 (PMC3709759; doi:10.3390/ijms140611842)
Supplement: Supplementary file 1 [file ijms-14-11842-s001.pdf]

Supplementary Information

Figure S1. CaCO3 crystals grown in presence of peptides AS8 and ES9 on flat wrinkle free substrate at three different pH values: LC-PolScope mode showing the birefringent retardance and orientation. The calcite does not show any defined morphology. Scale bar: 20 μm.

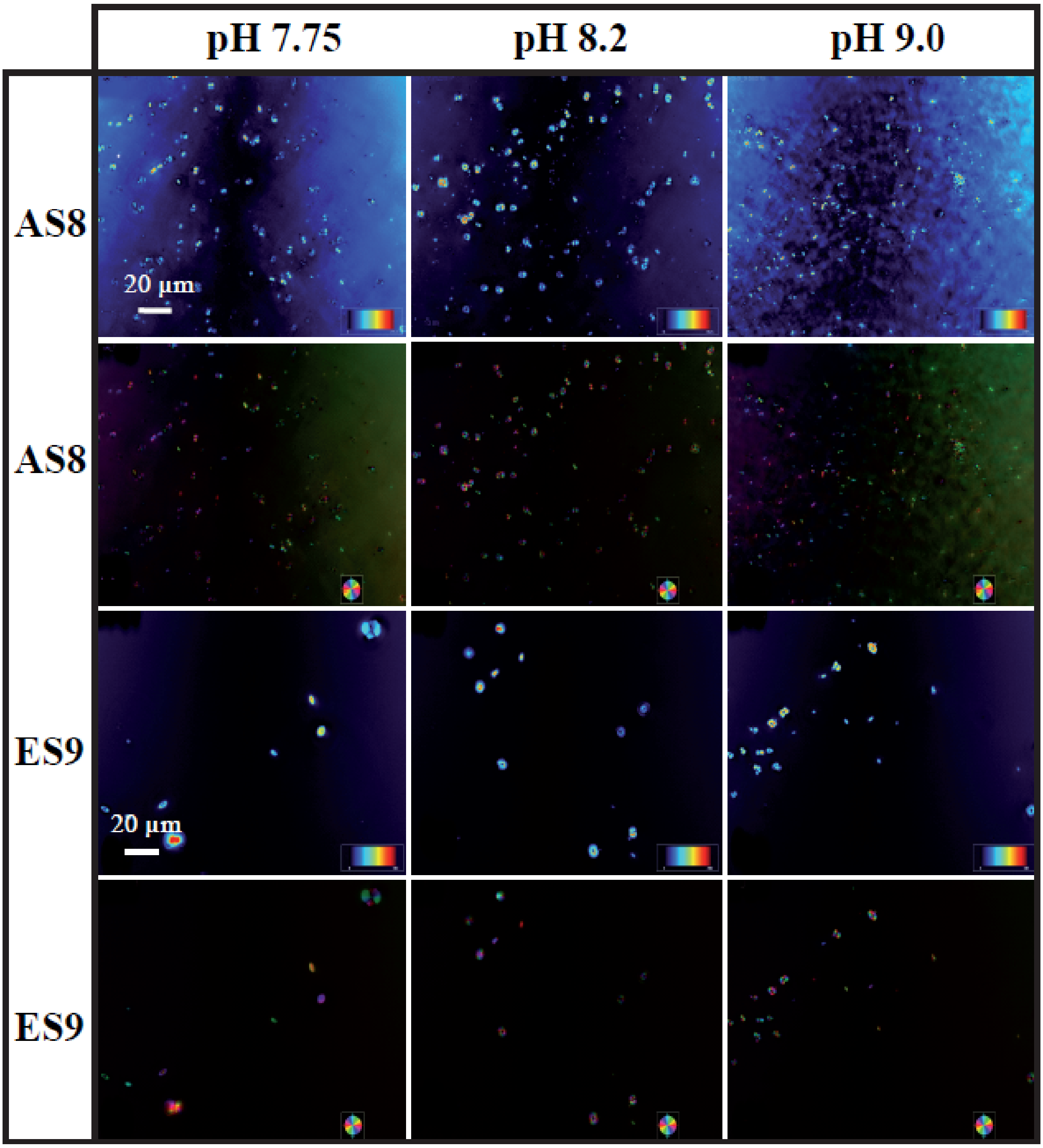

Figure S2. Raman spectra of calcite obtained in the presence of peptide AS8.

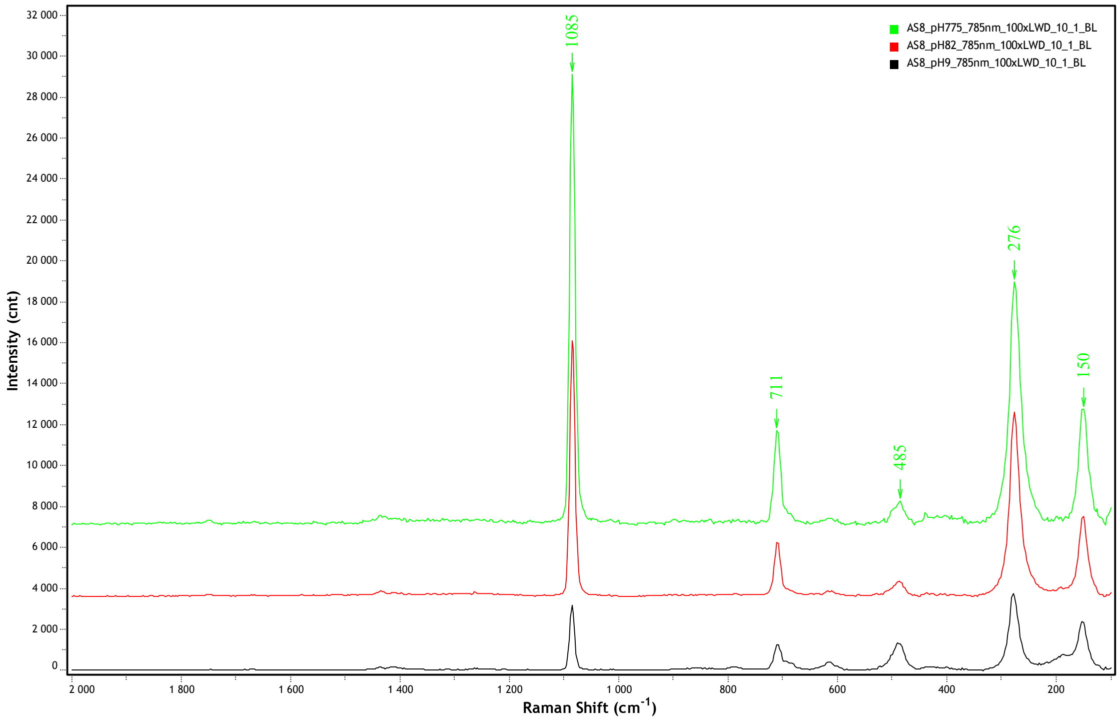

Figure S3. Raman spectra of calcite obtained in the presence of peptide ES9.

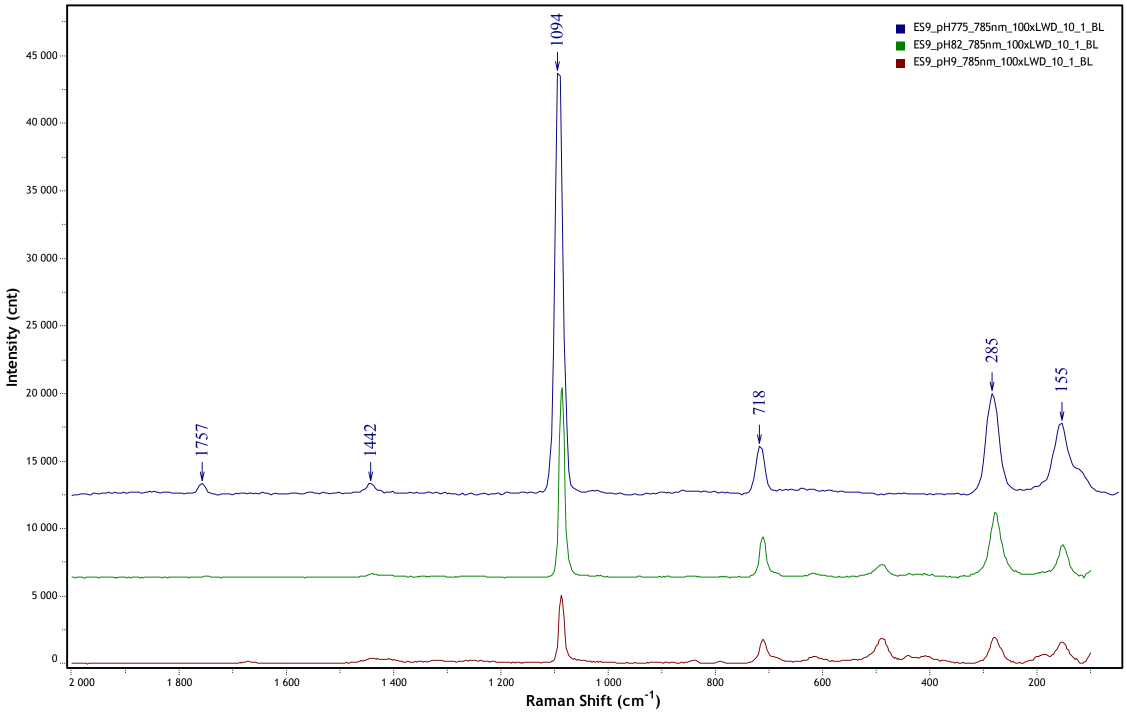

Figure S4. EDS spectra, SE image and elemental mapping of C, N, O, F, Si, Cl and Ca at *E* = 10 keV of peptide/Ca composites obtained under the influence of peptide AS8. (**a**) Peptide/Ca composites formed at pH 7.75; (**b**) Needle shaped peptide crystal formed at pH 8.2.

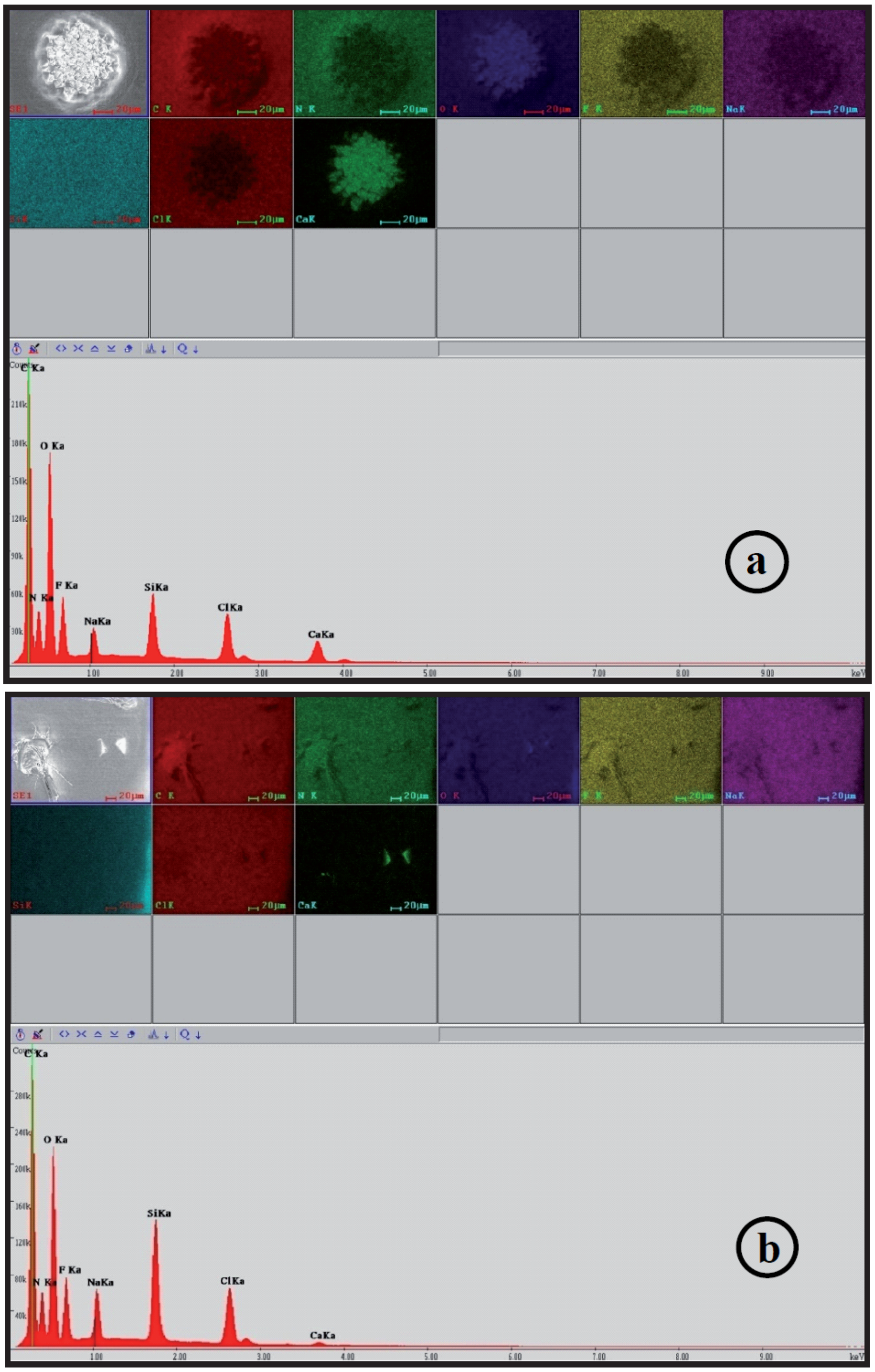

© 2013 by the authors; licensee MDPI, Basel, Switzerland. This article is an open access article distributed under the terms and conditions of the Creative Commons Attribution license (http://creativecommons.org/licenses/by/3.0/).
